# Etiology of severe invasive infections in young infants in rural settings in sub-Saharan Africa

**DOI:** 10.1371/journal.pone.0264322

**Published:** 2022-02-25

**Authors:** Estomih Mduma, Tinto Halidou, Berenger Kaboré, Thomas Walongo, Palpouguini Lompo, Justine Museveni, Joshua Gidabayda, Jean Gratz, Godfrey Guga, Caroline Kimathi, Jie Liu, Paschal Mdoe, Robert Moshiro, Max Petzold, Jan Singlovic, Martine Guillerm, Melba F. Gomes, Eric R. Houpt, Christine M. Halleux

**Affiliations:** 1 Haydom Research Center, Haydom Lutheran Hospital, Haydom, United Republic of Tanzania; 2 Institut de Recherche en Sciences de la Santé (IRSS), Clinical Research Unit of Nanoro (CRUN), Nanoro, Burkina Faso; 3 Division of Infectious Diseases and International Health, University of Virginia, Charlottesville, Virginia, United States of America; 4 School of Public Health and Community Medicine, Institute of Medicine, University of Gothenburg, Gothenburg, Sweden; 5 UNICEF/UNDP/WB/WHO Special Program for Research & Training in Tropical Diseases (TDR), World Health Organization, Geneva, Switzerland; National and Kapodistrian University of Athens, GREECE

## Abstract

**Background:**

Serious invasive infections in newborns are a major cause of death. Lack of data on etiological causes hampers progress towards reduction of mortality. This study aimed to identify pathogens responsible for such infections in young infants in sub-Saharan Africa and to describe their antibiotics resistance profile.

**Methods:**

Between September 2016 and April 2018 we implemented an observational study in two rural sites in Burkina Faso and Tanzania enrolling young infants aged 0–59 days old with serious invasive infection. Blood samples underwent blood culture and molecular biology.

**Results:**

In total 634 infants with clinical diagnosis of serious invasive infection were enrolled and 4.2% of the infants had a positive blood culture. The most frequent pathogens identified by blood culture were *Klebsiella pneumonia* and *Staphylococcus aureus*, followed by *Escherichia coli*. Gram-negative isolates were only partially susceptible to first line WHO recommended treatment for neonatal sepsis at community level. A total of 18.6% of the infants were PCR positive for at least one pathogen and *Escherichia coli* and *Staphylococcus aureus* were the most common bacteria detected. Among infants enrolled, 60/634 (9.5%) died. Positive blood culture but not positive PCR was associated with risk of death. For most deaths, no pathogen was identified either by blood culture or molecular testing, and hence a causal agent remained unclear. Mortality was associated with low body temperature, tachycardia, respiratory symptoms, convulsions, history of difficult feeding, movement only when stimulated or reduced level of consciousness, diarrhea and/or vomiting.

**Conclusion:**

While *Klebsiella pneumonia* and *Staphylococcus aureus*, as well as *Escherichia coli* were pathogens most frequently identified in infants with clinical suspicion of serious invasive infections, most cases remain without definite diagnosis, making more accurate diagnostic tools urgently needed. Antibiotics resistance to first line antibiotics is an increasing challenge even in rural Africa.

## Introduction

Children are at highest risk of dying in their first month of life- the risk being consistently greatest (36%) within the first 24 hours after birth, 37% during the remaining first week of life, and 28% for the first month across regions [1, 2]. Although child mortality has halved since 1990, progress in neonatal mortality is slow in highest-burden countries, and especially in Africa which is the only region with no decline in neonatal mortality between 1990 and 2019 [3] leading to predictions that most sub-Saharan African countries will miss their Sustainable Development Goal target for neonatal mortality [4]. In addition to immediate mortality, survivors of severe invasive neonatal infections are at risk of long-term neurodevelopmental impairment and disability with excess mortality after the neonatal period [5].

Infections are responsible for approximately 37% of the 2.9 million neonatal deaths in sub-Saharan Africa [6] and although the management of severe infections can reduce deaths by 84%, it is calculated that successful management has the greatest number of bottlenecks [7]. Rapid detection and immediate treatment are essential to save life. There are an estimated 6.9 million annual serious neonatal bacterial infections needing treatment, and one-third occur in sub-Saharan Africa, despite evidence of 25% reductions in all-cause neonatal mortality with immediate antibiotic treatment [8, 9]. The microbiological etiology of infections in young infants has been described in very few studies, mostly hospital based [10–13]. A study in African infants under 2 months of age brought to facilities showed that probable or definite pathogens in children requiring admission were identified in 10.6% of patients [10]. In a large multi-country Asian study, only 2.1% of blood cultures (102/4859) were able to identify a responsible pathogen [14]. A recent systematic review and meta-analysis of invasive bacterial infections in neonates found *Staphylococcus aureus*, *Klebsiella* spp., *Escherichia coli*, the most frequent pathogen identified in culture positive sepsis in sub-Saharan Africa [15].

Information on the burden of antibiotic resistance contributing to poor outcomes in neonates is lacking. Assessing the resistance profile of pathogens is important to ensure optimal treatment, avoid excessive and unnecessary antibiotic use and prevent further increases in antimicrobial resistance. Blood culture, the gold standard for diagnosis, has several limitations. Limited blood volume sampled in young infants, intermittent bacteriemia, and prior use of antibiotics in the mother at delivery can reduce blood culture yield. Consequently, molecular diagnosis is seen as a promising alternative for pathogen identification.

We conducted an observational study to identify microbiological causes of severe invasive infection in young African infants, using blood culture and molecular testing to identify possible causal pathogens. We also documented antibiotic resistance profiles for pathogens identified by blood culture. Finally, we assessed the association of clinical signs and symptoms, alone or in combination, with episode outcome.

## Methods

### Study design and participants

This was an observational study implemented between 2016 and 2018 in two rural sites in sub-Saharan Africa to identify pathogens causing severe invasive infections in young infants aged 0–59 days old.

In Burkina Faso the study took place in a rural catchment area of approximately 62,000 inhabitants, a demographic surveillance site served by 8 peripheral healthcare centers at which a nurse is located, and by the district hospital Centre Médical avec Antenne Chirurgicale Saint Camille de Nanoro, located in Central West Burkina Faso. Within the catchment area, the “Agents de Santé Communautaire” trained by the District Sanitaire (part of the Ministry of Health) followed newborns.

In Tanzania, the study took place in Haydom Lutheran Hospital (HLH) catchment area, situated in the north-central part of Tanzania, approximately 300 km from Arusha the nearest city and covering 74 villages and a population of 295,581 persons. The population in this catchment receives regular reproductive, clinical and nutritional care provided by the HLH Reproductive and Child Health Section (RCHS) through monthly mobile outreach clinics.

### Community-based surveillance of pregnancy and newborns

Prior to study initiation, surveillance in the communities was strengthened to enable all pregnancies to be identified. Eligible participants were pregnant women and live-born babies in the community catchment area. Consenting pregnant women were registered during their third trimester of pregnancy by Field Workers (FWs) or Community Health Workers (CHWs) or nurses trained to assess and manage young infants with signs of invasive infection using the young infant component of Integrated Management of Childhood Illness (IMCI) training package. FWs and CHWs conducted home visits to support antenatal care, help women prepare for birth, encourage skilled delivery and motivate the family to follow optimal newborn care practices immediately after birth. They also followed all women and newborns (by phone or home-visits) as early as possible after delivery. After birth, the mother-child pairs were visited at home several times until the baby reached 2 months old usually on days 1 (or as close as possible to the date of delivery), 2, 3, 5, 7, 10, 15, 21, 28, 42, 59, following WHO/UNICEF recommendations on newborn surveillance. Any young infant identified with danger signs was to be referred to the hospital for further assessment and possible enrollment.

### Hospital surveillance

Young infants born at the hospital and newborns presenting spontaneously to the referral hospital, clinically diagnosed with severe invasive infection were invited to participate in the study, on condition that the child was living in rural area and could be followed up at home after hospital discharge. Initially, only sick babies aged 0–7 days meeting inclusion criteria were invited but as recruitment progressed, the age criterion was expanded to include babies 0–59 days.

### Hospital enrollment

Infants aged 0–59 days, presenting with clinical suspicion of invasive infection were enrolled either at the Centre Médical Saint Camille de Nanoro, the referral hospital of Nanoro district in Burkina Faso, or the Centre Médical Saint Louis de Temnaoré, another district hospital, or at Haydom Lutheran Hospital (HLH) the referral hospital for 3 districts and 2 regions in Tanzania. Signs and symptoms used to identify clinical suspicion of invasive infections were any of the following: history of difficult feeding, movement only when stimulated, fever (axillary temperature ≥ 37.5 C) or low body temperature (axillary temperature < 35.5 C), respiratory rate > 60 breaths per minute, severe chest indrawing and history of convulsions. If informed consent was declined, infants were excluded.

### Sample size for hospital enrolment of neonates with severe invasive infections

The primary objective of the study was to describe the etiology of invasive severe infections in infants < = 59 days of age. We aimed to enroll 300 infants at each site (600 infants in total)—of which at least 50% were to be aged 0–7 days. It was anticipated that this number of children with an equal proportion of children in each age group would have a 95% CI of +/- 4% and based on a minimum of 5% positive rate in blood culture estimated from previous literature [10, 16], might yield 30 positive blood cultures results.

### Data collection and study procedures

At the hospital, detailed data on medical history, date and mode of delivery, prematurity, delivery complications, and use of antibiotics by the mother during labor were systematically collected in addition to clinical presentation including temperature, weight, height, appearance/coloration, respiratory rate, pulse, oxygen saturation, level of consciousness, chest indrawing, signs of dehydration, cord infection, tonus and evident birth defects. An abdominal, liver, spleen and chest examination was carried out at enrolment and daily if the child was admitted.

Results of laboratory assessments carried out (e.g. haemoglobin, white blood cells, or C-reactive protein measurement, culture of cerebrospinal fluid or urine culture), and treatment administered at the clinician’s discretion, were recorded. Any information on clinical evaluation and treatments provided were recorded on the Case Record Form. No attempt was made to influence clinical management and treatment which followed the local standard of clinical care. Use of antibiotics prior to hospital admission was not recorded.

All admitted patients were monitored daily during hospitalization until discharge and then at 7 and 28 days after discharge to ascertain clinical outcome, defined as either death or survival.

### Collection of samples

The study protocol allowed for study physicians to obtain blood samples on all infants with suspected severe invasive infection, irrespective of whether admission was later declined. Blood samples were taken upon enrollment and before treatment when possible, after a consultant neonatologist had trained clinical and laboratory staff on neonatal sampling. Up to 2 ml (for infants 0–7 days old), or up to 2.5 ml (for infants 8–59 days) venous blood was collected following standard procedures in place in hospital. Hemoglobin and glucose were measured upon admission on capillary blood, and venous blood collected for immediate blood culture and later PCR analysis. Blood samples were collected on EDTA tubes for molecular testing and stored at -20°C or—80°C until analysis.

In Burkina Faso, as the area is hyperendemic for malaria, malaria rapid tests (HRP-2 based antigen test) and microscopy (for patients with rapid test positive results) was performed upon enrollment.

In some cases, following routine patient care procedures, other blood testing (e.g. full blood count, C-reactive protein) were collected upon physician’s judgement, although not required as part of the protocol.

### Laboratory procedures—Samples analysis—Processing of samples

#### Blood culture

Automated blood culture (blood culture systems BD BACTEC Becton Dickinson and Company, Sparks, Maryland, USA BD Diagnostics Franklin Lakes, NJ, USA or BacT/Alert, BioMérieux, Marcy l’Etoile, France) was used to isolate bacterial pathogens. Up to 1.5 ml of blood was inoculated for culture. Positive samples were examined by Gram stain and sub-cultured onto Eosin Methylen Blue (EMB) (BioMérieux, Marcy l’Etoile, France) and Columbia (BioMérieux) + 5% sheep blood agar. Bacterial strains were identified with conventional microbiological tests. Bacillus spp. and coagulase-negative staphylococci were considered contaminants.

*Antimicrobial susceptibility testing* for the isolated pathogens was done via disk diffusion. Broth from positive culture bottle was inoculated on Mueller-Hinton plates, antibiotic disks selected upon interpretation of Gram staining, and results were read after 18h incubation. Tested antibiotics included those specified in the protocol in addition to those routinely tested according to national guidelines. Interpretation of results was based on Clinical and Laboratory Standards Institute (CLSI) breakpoints [17]. Detection of methicillin resistant staphylococcus aureus was conducted using cefoxitin disks. Quality control strains included ATTC Strains *E*.*coli* 25922 and *S*. *aureus* 25923.

#### Molecular testing

Nucleic acid extraction was conducted prior molecular testing. Total nucleic acid from blood was extracted using the High pure viral nucleic Acid large volume kit (Roche, Wilmington, MA) [18]. Molecular testing was done by real-time polymerase chain reaction (PCR) with a customized TaqMan Array Card on ViiA 7 Real time PCR system (Life Technologies, Carlsbad, CA) to detect 16 bacterial, 1 protozoal and 4 viral pathogens [18, 19]. The following pathogens were included: *Acinetobacter baumannii*, *Escherichia coli/Shigella spp*., *Enterococcus faecalis*, *Haemophilus influenzae*, *Klebsiella pneumoniae*, *Listeria monocytogenes*, *Mycobacterium tuberculosis*, *Neisseria meningitidis*, *Pseudomonas aeruginosa*, *Streptococcus agalactiae*, *Staphylococcus aureus*, *Streptococcus pneumoniae*, *Streptococcus pyogenes*, *Salmonella spp*., *Ureaplasma spp*., *Cytomegalovirus*, *Enterovirus*, *Herpes Simplex Virus 1*, *Herpes Simplex Virus 2*, and *Plasmodium spp*. (S1 Appendix). Cards were designed and validated by the University of Virginia. Molecular testing for both sites was performed at the Research Center of the Haydom Lutheran Hospital. Data were analyzed with ViiA 7 real time PCR software version 1.2.4. Any quantification cycle (Cq) result of <35 was considered positive. External controls, MS2 and PhHV were spiked into each sample to monitor the extraction and amplification efficiency. One extraction blank was included per batch of extraction to rule out lab contamination. While *K*. *oxytoca* was included in the pathogens to be detected by TaqMan, recent data have highlighted the potential for *K*. *oxytoca* to represent a contaminant, and hence *K*. *oxytoca* has been considered as not clinically significant for the final analysis and therefore excluded for the analysis.

#### Hemoglobin testing

Measurement of capillary blood was tested using Hemoccue Hb 201 DM system or hematology automate (automatic hematology analyzer Swelab or Sysmex XN-1000).

Glucose testing: quantitative determination of glucose in capillary blood was performed using the HemoCue Glucose 201+ analyser.

Quality assurance activities for both study sites included specific training for blood sample collection from neonates, review of algorithms for pathogen identification, and participation in an external quality assessment (EQA) scheme. The EQA was based on the use of proficiency panels (made of lyophilized strains) sent to the sites before the initiation of the study, and once during the recruitment period. The EQA for Diagnostic Bacteriology scheme is organized and managed by the Unit of Tropical Laboratory Medicine from the Institute of Tropical Medicine in Antwerp in Belgium. Both sites were found to be proficient. Quality control data for each technique were recorded and monitored.

### Consent and ethical approval

Written informed consent was obtained from parents/caretakers of young infants for each participant, through signature or thumbprint in the presence of a literate witness when indicated. The study was approved by WHO Ethics Review Committee and the relevant national ethics committee (Comité d’Ethique pour la Recherche en Santé (No 2016-7-085) and Comité d’Ethique Institutionnel pour la Recherche en Sciences de la Santé (N/Réf. A02-2016/CEIRES)) in Burkina Faso and the National Institute for Medical Research (NIMR) Ethical Committee and Ministry of Health Community Development, Gender, Elderly and Children (NIMR/HQ/R.8c/Vol.1/426) in Tanzania.

### Statistical analysis

Prior to analysis a Data Review Committee was convened with terms of reference to adjudicate causes of deaths on whether these were likely (or not) to have been due to invasive infection, to advise on the interpretation of the PCR results and their clinical significance, confirm “contaminants” in blood culture and advise on the cohort to be used for analysis. The committee adjudicated all deaths and advised that the full cohort should be used but stratified by severity sub-groups, confirmed that site-level determination of contaminants had been appropriate, and in the absence of a control group, advised separation of PCR analysis from blood culture analysis.

Frequencies and proportions are given for binary and categorical data. The number of observations, mean values and standard deviations are given for continuous data.

Fisher’s Exact tests were used for categorical data. Odds Ratios are presented together with two-sided 95% CI. T-test was used for continuous data. The level of significance was set at a p value <0,05. Missing data were not imputed. Stata 16.1 (Stata Corp., TX, USA) was used for analysis.

The original protocol specified that microbiological confirmation by composite criteria of positive blood culture or cerebrospinal fluid culture and/or positive result for real time PCR assays (on blood samples) for selected pathogens would be used as the primary endpoint. However, after study start, new evidence emerged from studies [14, 20] with healthy controls of PCR positivity for some pathogens even in healthy controls. Consequently, the results of blood culture and PCR in blood were not combined and the main analysis reported here separated results obtained from blood culture and PCR. For PCR, only samples with 0.5 ml of blood or more were included.

Outcome parameters were evaluated through hospital duration and mortality. Mortality data are presented as in-hospital mortality prior to discharge and post-discharge mortality occurring either in community or upon readmission.

## Results

In total 2,429 pregnant women were followed until term. Pregnancy outcomes were collected for 2,281 (93.9%) pregnancies accounting for 2,291 births: there were 2,174 (94.9%) livebirths, 74 (3.2%) stillbirths, and 21 (0.9%) miscarriages; in 22 (1.0%) cases, information was not available to differentiate between stillbirths and miscarriages (S1 Fig). A further 575 liveborn babies (whose mothers were not enrolled during pregnancy) were enrolled directly after birth. Thus altogether 2,749 newborns were enrolled as part of community surveillance and most (97%) were followed up regularly for 59 days. Of these surveillance newborns, 361 were enrolled in the hospital part of the study with clinical suspicion of invasive infection. In addition, 273 newborns with clinical assessment of invasive infection were enrolled when assessed at the health facility (S2 Fig).

### Study population

Altogether 634 infants with clinical suspicion of invasive infection were enrolled at hospital between 29 September 2016 and 13 April 2018. Demographic data are presented in Tables 1 and 2. Fifty-four (54%) of the enrolled infants were aged 0–7 days, and 46% were aged 8 to 59 days. Most enrolled infants were born at a health facility (86%) while the remainder (14%) were born at home. Birth weight was available for 468 (74%) infants, and among them, 106 (23%) had a low birth weight (defined as birth weight < 2500 gr). APGAR score at 5 minutes was reported in 432 infants (68%); among them, 25% had an APGAR score of <9 at 5 minutes. A small percentage (16%) of the mothers reported having received antibiotics before delivery.

**Table 1:** Demographics characteristics of young infants with clinically suspected invasive infection.

Characteristic	(n)	Burkina Faso	Tanzania	All
		n (%)	n (%)	n (%)
Gender	634			
Boys		167 (50.3)	178 (58.9)	345 (54.4)
Girls		165 (49.7)	124 (41.1)	289 (45.6)
Age—distribution per age group	634			
0–7 days		147 (44.3)	198 (65.6)	345 (54.4)
8–59 days		185 (55.7)	104 (34.4)	289 (45.6)
Gestational age at birth	634			
Term		314 (94.6)	263 (87.1)	577 (91.0)
Premature		16 (4.8)	29 (9.6)	45 (7.1)
Unknown		2 (0.6)	10 (3.3)	12 (1.9)
Birth weight^a^	468			
< 2500 g		67 (23.2)	39 (21.8)	106 (22.6)
≥ 2500 g		222 (76.8)	140 (78.2)	362 (77.4)
Apgar score at 5 minutes^b^	432			
≥ 9		204 (73.9)	120 (76.9)	324 (75.0)
< 9		72 (26.1)	36 (23.1)	108 (25.0)

^a^ For 166 infants, birthweight was not available.

^b^ For 202 infants, Apgar score at 5 minutes was not available.

**Table 2:** Characteristics of mothers of young infants with clinically suspected invasive infection.

Characteristic	Burkina Faso	Tanzania	All
	n (%)	n (%)	n (%)
Total number of babies enrolled	332	302	634
Place of delivery			
Health facility	320 (96.4)	224 (74.2)	544 (85.8)
Home	12 (3.6)	78 (25.8)	90 (14.2)
Mode of delivery			
Vaginal delivery without instruments	293 (88.3)	235 (77.8)	528 (83.3)
Vaginal delivery with vacuum	1 (0.3)	0 (0)	1 (0.2)
Caesarean section elective	1 (0.3)	3 (1.0)	4 (0.6)
Caesarean section emergency	37 (11.1)	64 (21.2)	101 (15.9)
Antibiotics taken by mother before delivery			
Yes	29 (8.7)	70 (23.2)	99 (15.6)
No	288 (86.7)	201 (66.6)	489 (77.1)
Unknown	15 (4.5)	31 (10.3)	46 (7.3)

### Clinical characteristics

At hospital, all sick infants enrolled presented one or more of the criteria for clinical suspicion of severe invasive infection: fever or hypothermia (79%), history of difficult feeding (33%), elevated respiratory rate > 60 per minute (23%), severe chest indrawing (11%), movement only when stimulated (9%) and history of convulsions (8%).

When clinically examined, about 2/3 of the infants had fever, and 11% presented with hypothermia (axillar temperature < 35.5°C). Only 3% had tachycardia (defined as > 180 beats per minute), but 26% had increased respiratory rate and 23% had low oxygen saturation. Altered consciousness was present in 12%, and coma in 1% (Tables 3 and 4).

**Table 3:** Presentation of criteria for clinical suspicion of severe invasive infection at time of presentation at the hospital.

	Burkina Faso	Tanzania	All
	n (%)	n (%)	n (%)
Total	332	302	634
**Criteria**			
History of difficult feeding	30 (9.0)	178 (58.9)	208 (32.8)
Movement only when simulated	26 (7.8)	33 (10.9)	59 (9.3)
Temperature < 35.5°C or ≥ 37.5°C	314 (94.6)	189 (62.6)	503 (79.3)
Respiratory rate > 60 breaths /minute	29 (8.7)	119 (39.4)	148 (23.3)
Severe chest indrawing	33 (9.9)	39 (12.9)	72 (11.4)
History of convulsions	8 (2.4)	41 (13.6)	49 (7.7)

**Table 4:** Clinical presentation of young infants with clinically suspected invasive infection (clinical examination upon arrival at the hospital).

Characteristic	n (%)
Total	634
Temperature, °C	
< 35.5	68 (10.7)
35.5–37.4	139 (21.9)
≥ 37.5	427 (67.4)
Heart rate	
≤ 180 beats/minute	594 (93.7)
> 180 beats/minute	18 (2.9)
Unknown	22 (3.5)
Respiratory rate	
≤ 60 breaths/minute	454 (71.6)
> 60 breaths/minute	163 (25.7)
Unknown	17 (2.7)
SpO2 ^a^	
≥ 92%	473 (74.6)
< 92%	146 (23.0)
Unknown	15 (2.4)
Status of consciousness	
Altered	73 (11.5)
Coma	6 (0.9)
Chest indrawing	124 (16.9)
Grunting	69 (10.9)
Nasal flaring	71 (11.2)
Severe dehydration	7 (1.1)
Cord infection	50 (7.9)
Tonus	
Floppy	86 (13.6)
Stiff	6 (0.9)
Fontanel	
Bulging	9 (1.4)
Depressed	6 (0.9)

^a^ SpO2: Oxygen saturation.

### Laboratory findings

Results of white blood cells count were available for 500 infants, the average value being 12.5x9/L. Fifty (10%) babies had a white blood cells count of > 20x9/L and 18 (3.6%) had leukopenia (white blood cells count < 5x9/L). Glycemia upon admission are available for 217/634 (34%) children, among whom 18 (8%) had a blood glucose < 40 mg/dL.

#### Detection of pathogens

*Blood culture*. Blood cultures were not performed in 16 (2.5%) of enrollees. Out of the 618 blood cultures performed, 87 were positive, of which 61 grew organisms classified as contaminants (S1 Table). The remaining 26 blood culture positive grew organisms classified as clinically relevant, corresponding to a proportion of lab-confirmed invasive infection per suspected episode of 4.2% (Table 5). For the purpose of the analysis, only blood cultures positive for pathogens considered as clinically relevant were considered positive. Blood culture positivity rates were slightly different between sites (5.6% in Tanzania vs 2.8% in Burkina Faso) (Table 5). Overall age-specific positivity rate for blood culture was 5.3% (18/338) in infants aged 0–7 days versus 2.9% (8/280) in infants aged 8–59 days (RR 1.86, 95% CI: 0.82–4.22; *p* = 0.13, not significant).

**Table 5:** Hemoculture results and positivity rate of quantitative polymerase chain reaction (PCR) detection in blood in young infants with clinically suspected invasive infection.

Criteria	Burkina Faso	Tanzania	All
	n (%)	n (%)	n (%)
Infants with hemoculture sample processed ^a^	317	301	618
Infants with positive hemoculture results	40 (12.6)	47 (15.6)	87 (14.1)
Infants with hemoculture positive for microorganisms regarded as pathogens	9 (2.8)	17 (5.6)	26 (4.2)
Infants with PCR result available ^b^	283	201	484
Infants with PCR result negative	214 (75.6)	180 (89.6)	394 (81.4)
Infants with PCR result positive for at least one pathogen	69 (24.3)	21 (10.4)	90 (18.6)
Infants with PCR result positive for more than one pathogen	10 (3.5)	2 (1.0)	12 (2.5)
Infants with result PCR positive for at least one bacteria	38 (13.4)	15 (7.5)	53 (11.0)

^a^ No hemoculture sample was processed in 16 infants.

^b^ PCR results in blood were only available for 484 infants. In 130 infants no blood sample was available, in 16 infants the volume of blood sample was <0.5 ml, and in 4 infants the external controls had failed and therefore the results of PCR analysis were considered not valid.

Volume of blood based on weight after sampling showed a trend towards better yield with more volume, but the trend was not statistically significant.

Separation of infants who received systemic antibiotics after sampling from those who received antibiotics before sampling shows a slightly higher, non-significant hemoculture positivity rate in infants with no antibiotics before sampling of 5.1% (12/237) vs 4.2% (14/330) in infants who received antibiotics before sampling (RR 1.19, 95% CI: 0.56–2.53; *p* = 0.65, not significant).

No infant had more than one clinically relevant pathogen identified via blood culture. Gram negative organisms were found in 15/26 (58%) of positive cultures. *Klebsiella pneumonia* and *Staphylococcus aureus* (8/26 of blood culture positive each) were the most frequent pathogens identified, followed by *Escherichia coli* (4/26 of blood culture positive). Organism type found differed between sites, with all cases of *Klebsiella pneumoniae* and 6/8 cases of *Staphylococcus aureus* found in Tanzania, and 3/4 cases of *Escherichia coli* found in Burkina Faso (Table 6). All 8 cases of *Klebsiella pneumoniae* were all found in very young infants aged 0–6 days.

**Table 6:** Pathogens detected in hemoculture of young infants with clinically suspected invasive infection.

Bacteria	Burkina Faso	Tanzania	All
	n	n	n (% of samples positive for the bacteria among babies with hemoculture positive)
All	9	17	26
*Escherichia coli*	3	1	4 (15.4)
*Group A Streptococcus*	1	0	1 (3.8)
*Klebsiella pneumoniae*	0	8	8 (30.8)
*Pseudomonas aeruginosa*	1	0	1 (3.8)
*Serratia marcescens*	0	1	1 (3.8)
*Staphylococcus aureus*	2	6	8 (30.8)
*Streptococcus pneumoniae*	1	0	1 (3.8)
*Streptococcus sp*.	1	0	1 (3.8)
Gram-negative rod, uncertain significance	0	1	1 (3.8)

Only one infant had a CSF culture performed, with negative results. Two infants had urine cultures performed, both negative.

*Molecular testing*. A blood sample was available for molecular testing in 556 (87.7%) of the infants and valid results were obtained in 484 infants (76.3%). Amongst infants with valid PCR results, 90/484 (18.6%) infants were PCR positive for at least one pathogen and 12/484 (2.5%) were positive for more than one pathogen (Table 5).

*Escherichia coli* and *Staphylococcus aureus* were the most common bacteria detected by PCR (6.8% and 1.7% respectively). Cytomegalovirus and enterovirus, positive in 4.1% and 2.9% of the samples respectively, were the most common viruses detected, and *Plasmodium* spp, was also found as protozoa but only in Burkina Faso (2.1% of the samples) (S2 Table).

*Correlation between pathogens identified by blood culture vs molecular testing*. Out of the 26 infants with clinically relevant pathogens isolated from blood culture, 16 (61.5%) also had blood samples analyzed by PCR. Of these 16, 8 had a bacteria identified in blood by PCR, and in all 8 cases, the pathogen identified by PCR was the same as that identified in blood culture (S3 Table). Culture and PCR detected 8 bacteria, culture detected an additional 8 bacteria missed by PCR, and PCR detected an additional 46 bacteria in infants with blood culture negative. The quantities of bacteria detected by qPCR as measured by cycle threshold in the 8 culture positive specimens were 31.8+/- 3.9 versus 32.3+/-2.5 in the 46 culture negative specimens, p = NS.

*Antibiotic susceptibility and resistance profile*. Gram-negative isolates were only partially susceptible to first line WHO recommended treatment for neonatal sepsis at community level (penicillin, ampicillin and/or gentamicin). *Klebsiella pneumoniae* was systematically resistant to ampicillin and gentamicin and also to chloramphenicol and ceftriaxone; the isolates remained susceptible to other aminoglycosides, fluoroquinolones and nalidixic acid. Gram-positive pathogens were usually sensitive to gentamicin and ceftriaxone. No strain of *Staphylococcus aureus* was found methicillin resistant (S4 Table).

*Parasitology*—*Malaria detection*. 510 out of the 634 (80.4%) children enrolled were tested with a rapid malaria diagnosis test. In Burkina Faso, malaria was diagnosed in 6 (1.8%) of babies via rapid test, confirmed via microscopy. The infants positive for malaria were all aged above 30 days and 5 out of 6 had a blood sample taken for PCR in blood and 4 had a sample positive for plasmodium. PCR was positive for Plasmodium in 6 additional cases. No malaria positive cases were reported in Tanzania.

### Antibiotic treatment

Among enrolled infants, 93.1% received systemic antibiotics for the suspected infection. The treatment regimen followed local common practice and differed slightly between sites. About half (51.6%) of the sick infants received the first dose of systemic antibiotics before sampling.

In Burkina Faso, for those who received systemic antibiotics, ceftriaxone was the most frequently prescribed antibiotic (54.3% of cases), often prescribed with gentamicin. In Tanzania, 97.3% of the young infants received gentamicin usually associated with ampicillin, and only 9.0% of the infants received ceftriaxone. Frequency of systemic antibiotic treatment is provided in (S5 Table).

### Hospital stay and discharge after hospitalization

There were 528/634 hospital admissions. In Tanzania, all babies except one were hospitalized while in Burkina Faso 105 babies were not admitted (S2 Fig). The average number of days in hospital was therefore higher in Tanzania than in Burkina Faso (6.3 days vs 2.4 days respectively). Having a positive blood culture slightly increased duration of hospital stay in Tanzania (average stay in hospital of 6.9 days vs 6.2 days for babies with blood culture positive vs negative) and made no difference in Burkina Faso (average stay in hospital of 2.4 days in both groups). Out of the 528 babies hospitalized, 50 (9.5%) babies died during hospitalization. 441 (83.5%) were discharged at home upon improvement of their condition or recovery while 12 (2.3%) and 25 (4.7%) were discharged against medical advice or referred to another medical structure respectively (S2 Fig).

#### Follow up after discharge

Out of the 584 babies alive at hospital exit, 554 (94.8%) patients were followed up at least once thereafter: 516 infants (88.4%) had fully recovered but 10 babies died, 6 of whom had been referred to another health structure, 2 who were discharged against medical advice and 2 who worsened post-discharge. A total of 28 infants were followed up but had not fully recovered when follow up ended after 4 weeks or more post discharge (S2 Fig).

### Mortality rate in infants with clinical suspicion of invasive infection

Among 634 infants enrolled, 60 (9.5%) died, the majority (48/60, 80%) in the youngest age group, reflected in the observed case fatality ratio of 13.9% vs 4.2% for the two age groups (Table 7). For most deaths, no pathogen was identified either by blood culture or molecular testing, and hence a causal agent remained unclear. Among the 60 deaths, only 8 (13.3%) had positive blood culture (3 for *Klebsiella pneumoniae*, 2 for *E*. *coli*, 1 for *Pseudomonas aeruginosa*, 1 for *Streptococcus pneumoniae* and 1 for *Streptococcus* spp.), and 9 (15.0%) were PCR positive in blood (2 for Cytomegalovirus, 2 for *Escherichia coli/Shigella*, 2 for *Staphylococcus aureus* (1 also being positive for *Pseudomonas aeruginosa*), 2 for *Streptococcus pneumoniae* and 1 for *Streptococcus pyogenes* (S6 Table). The observed case fatality ratio was higher in the group with blood culture positive compared with blood culture negative results: 30.8% vs 8.8% respectively (RR 3.51, 95% CI: 1.86–6.60; p = 0.0002). A positive result for molecular testing on blood was not associated with an increased mortality rate overall (Table 7). Infants PCR positive for bacteria did have higher mortality compared with PCR negative infants but this difference was not statistically significant.

**Table 7:** Mortality rate in infants with clinical suspicion of invasive infections.

Characteristic	Total, n	Death, n	Death, %
Infants enrolled (all)	634	60	9.5
Per age groups:			
Infants aged 0–7 days	345	48	13.9
Infants aged 8–59 days	289	12	4.2
Per groups based on laboratory results:			
Infants with positive blood hemoculture	26	8	30.8 ^a^
Infants with hemoculture negative	593	52	8.8
Infants with PCR in blood positive for a pathogen (any pathogen)	90	9	10.0
Infants with PCR in blood negative (no pathogen detected)	394	35	8.9
Infants with PCR in blood positive for a bacteria (any bacteria)	53	7	13.2 ^b^
Infants with PCR in blood negative for a bacteria (no bacteria detected)	431	37	8.6

^a^ Compared to infants with hemoculture negative: RR 3.51, 95% CI: 1.86–6.60; p = 0.0002.

^b^ Compared to infants with PCR negative for bacteria: RR 1.54, 95% CI: 0.72–3.28; p = 0.2693.

### Association of clinical signs and symptoms with outcome

The odds ratio for selected clinical signs and symptoms associated with death is reported in Table 8 adjusted for age group. Higher mortality rates were associated with low body temperature, tachycardia, respiratory symptoms, convulsions, history of difficult feeding, movement only when stimulated or reduced level of consciousness, diarrhea and/or vomiting.

**Table 8:** Adjusted odds ratio for selected clinical signs and symptoms being associated with death.

Clinical sign or symptom	Adjusted OR (95% CI) ^a^	p-value
Fever measured in hospital	0.40 (0.23–0.70)	0.001
Fever reported	0.34 (0.20–0.60)	<0.001
Low body temperature	5.91 (3.21–10.86)	<0.001
High respiratory rate and/or severe chest indrawing and/or low oxygen saturation	2.97 (1.69–5.24)	<0.001
Heart rate (high/low)	3.66 (1.23–10.91)	0.020
Convulsions	4.49 (2.29–8.80)	<0.001
History of difficult feeding	4.12 (2.34–7.26)	<0.001
Movement only when stimulated and/or reduced level of consciousness	13.83 (7.57–25.27)	<0.001
Diarrhoea and/or vomiting	5.33 (2.84–10.02)	<0.001

^a^ OR: odds ratio; CI: confidence interval.

Presented ORs are adjusted for age group.

## Discussion

The study was designed to provide information on pathogens in young infants with clinical presentation of invasive infections. We found an overall blood culture positivity rate of 4.2% in infants enrolled with clinical presentation of invasive infection although the positivity rate was higher (13.3%) in the infants who died. The low yield, despite attempts to shorten the time between sample collection and culture, corresponds with previous literature in a similar population, including the large multi-country ANISA study [14] but limits any correlation between antibiotics resistance, treatment administered and outcomes. A few studies have obtained 10–13% positivity rates [10, 12, 16] and even 37% [21] but these were studies generally conducted in infants qualifying for intensive care or where larger blood volumes were sampled for blood culture. Direct PCR on blood increased detection substantially, yielding a bacterial pathogen in ~11%, a viral pathogen in 7%, and Plasmodium in 2%. Therefore, like previous studies, it was not possible to attribute a definite causal pathogen for most patients, even those who died.

The study provides new insights on the role and limitations of current molecular diagnosis tools for laboratory confirmation of etiological causes of invasive infections. While molecular analysis yielded higher positive rates than blood culture (18.6% of positivity for molecular detection of pathogens in blood), the yield remained low for confirmation of clinical suspicion of sepsis. A major challenge of blood culture or quantitative PCR in neonates or young infants is the low sample volume which can safely or ethically be drawn. In our study we limited total sampling volume to that required for immediate management (haematology and biochemistry testing) and blood culture and molecular analysis to 2–2.5 ml, which meant that the volume retained for identifying pathogens was usually ≤ 1 ml. Larger blood draws might have improved sensitivity of blood culture and molecular analysis [16, 22, 23] and indeed, we noticed that infants with higher available blood had higher PCR detection rates, suggesting blood volume is an important determinant.

Molecular tests alone cannot yet be used to replace hemoculture and guide a therapeutic decision reliably, but they have become valuable in concert with other modalities to understand epidemiological causes of childhood illness and death. For example, the CHAMPS network uses molecular tests among many other diagnostic techniques to detect pathogens, however these data are combined with multiple other modalities to attribute final etiologies [24]. Clinical judgement remains essential as molecular tests may identify pathogens that have no clinical consequences [13]. Overall, while molecular testing has the potential to widen the diagnostic spectrum and provide rapid diagnosis compared to blood culture, PCR still has limitations in clinical practice in addition to cost.

As expected, the study confirms a concentration of deaths in the first week of life [3, 6, 14]. While admissions in the study were balanced between infants aged 0–7 days and 8–59 days, 48 out of 60 deaths occurred in infants aged 0–7 days at admission. Age at death was not the same as age of admission and 37/60 deaths occurred in the first week of life, 17/60 deaths occurred in infants aged 8–28 days, and only 6/60 deaths occurred in infants aged more 29 days, confirming the first week of life as particularly vulnerable for survival. Mortality in hospitalized infants with positive blood culture was 3 times higher than in infants with negative blood culture, confirming previous findings [12].

Although the sample size was small, the pathogens most frequently detected in blood culture were *Staphylococcus aureus*, *Klebsiella pneumoniae*, and *Escherichia coli*, corresponding with previous results from sub-Saharan Africa [12, 15]. Although both study sites were in rural areas, there were differences in pathogens. All *Klebsiella pneumoniae* were detected in Tanzania; this may either reflect a difference in geographical variation of pathogens or reflect the occurrence of a local outbreak since 6 out of 8 cases were detected over a period of three to four weeks. While the temporal presentation may reflect a cluster of cases, the cases included infants born in the hospital and at home, and it was not possible to identify any links between the cases.

The role of *Streptococcus agalactaie* seems relatively minor in our cohort. However, despite a careful follow up of pregnancies and newborns, some deaths occurred very prematurely at home for which we have no information, and therefore the importance of Group B *Streptococcus* may be underestimated by the lack of information on those cases of deaths at home before referral.

As per WHO recommendations when referral is not possible, empiric treatment for severe invasive infection can be initiated with amoxicillin or ampicillin plus gentamicin. While this treatment covers most infections, it was not appropriate for some gram-negative infections where resistance to ampicillin and gentamicin was considerable (e.g., 8/8 of *Klebsiella pneumonia* isolates resistant to ampicillin and gentamycin).

Standard treatment administered to sick infants varied between the two sites, with ceftriaxone being used more frequently in Burkina Faso (in about 50% of the cases treated with systemic antibiotics) than in Tanzania (in <10% of the cases), and gentamicin was used much less in Burkina Faso (42% of cases treated with systemic antibiotics) than in Tanzania (97% of cases). While ceftriaxone is not used as first line treatment in Tanzania, we detected high rates of resistance to this antibiotic for some pathogens, with 100% of the *Klebsiella pneumoniae* identified in Tanzania as drug-resistant. In general, antimicrobial resistance was important in Tanzania, where *Klebsiella pneumoniae* was resistant to several common antibiotics including ceftriaxone, suggesting extended spectrum beta lactamase use.

Respiratory symptoms, tachycardia, low body temperature, history of difficult feeding or reduced level of consciousness, but also diarrhea and/or vomiting and to a lesser extent fever were all associated with poorer outcomes. This is consistent with previous findings correlating symptoms with severe illness and poor outcomes in young infants [25]. Limited numbers did not permit a correlation analysis of signs and symptoms and blood culture positivity.

Several study limitations were identified. The inclusion and exclusion criteria captured a higher number of infants suspected to be severe invasive infections. However, they did not exclude important co-morbidities such as birth asphyxia, preterm birth or severe congenital anomalies which are related to neonatal morbidity [6, 26] and made it difficult to ascertain the role of a potential pathogen compared with other risk factors on the clinical presentation and outcome of these cases.

Secondly, the low yield of blood culture in identification of pathogens may have been influenced by the administration of antibiotics prior to blood sampling. As mentioned above, about half the enrolled infants received the first dose of antibiotics on admission, prior to sampling and antibiotic treatment prior to admission was not systematically documented.

Third, diagnosis is limited to blood culture and molecular testing on blood for specific pathogens. Other large studies [14] using additional sample types or diagnostic methods, were able to attribute the presumed bacterial infection to bacteria in 16% of the cases when using a combination of blood culture and molecular testing in blood and respiratory samples.

Fourth, the study did not have a control group to compare PCR results. This omission complicates the interpretation of the PCR results given the findings of prior studies [13, 14, 20] which have shown that certain target pathogens (such as CMV, *S*. *pyogenes*, *P*. *aeruginosa*, and *K*. *oxytoca*) were detected by molecular testing in blood of control infants without clinical symptoms. While the overall positivity rate of the PCR yield was higher than blood culture yield, it was also less strongly associated with poor outcomes. For example, infants that were culture positive for *E*. *coli* or *K*. *pneumoniae* showed a 42% mortality, while those that were PCR positive for these pathogens showed only a 5% mortality. It is plausible that this mortality difference is due to the detection of living versus dead bacteria. On the other hand, detection by PCR of *Pseudomonas aeruginosa*, *S*. *aureus*, *S*. *pneumo*, *and S*. *pyogenes* all showed a higher 20–50% mortality, arguing that these PCR detections are clinically significant.

Fifth, while the study, based on close follow up and integrated community case management (iCCM), was designed to rapidly detect clinical deterioration in newborns and neonates, some deaths occurred at home before hospital referral, particularly immediately after birth. We therefore missed information on a certain number of the neonatal deaths which occurred before hospital referral/admission could be achieved.

Finally, the sample size was small, and in addition to a low blood culture positivity rate and the lack of a control group for PCR testing, the number of laboratory-confirmed invasive infections for the whole study was low and limited analysis of blood culture results and clinical outcomes.

However, this is one of the very few reports of data on etiological causes of invasive infections in very young infants in rural sub-Saharan Africa. The study was conducted in remote areas, where efforts had been made to reflect what happens in rural communities and ensure that lab component of the study was optimally designed and implemented. The study was based on an extensive surveillance at community with rapid referral to hospital from the communities and blood sampling, handling, and culture occurring within a very short timeframe. The teams were fully trained for neonatal blood sampling and had research experience. This study provides hitherto missing data on etiological causes of infection and levels of antibiotic resistance in treating neonatal invasive infections in rural sub-Saharan Africa.

## Supporting information

S1 Appendix Pathogens included in the Taqman Array Card panel. (PDF)

S1 Fig Flow chart of surveillance of pregnancies and young infants in community. (PDF)

S2 Fig Flow chart of infants identified at hospital as clinical suspicion of invasive infection. (PDF)

S1 Table List of blood culture isolates determined as clinically non-significant. (DOCX)

S2 Table Pathogens detected by quantitative polymerase chain reaction in blood of young infants with clinically suspected invasive infection. (DOCX)

S3 Table Correlation between pathogens found in blood culture and PCR. (DOCX)

S4 Table Antibiotic susceptibility and resistance profile of bacteria isolated in hemoculture. (DOCX)

S5 Table Frequency of different systemic antibiotics administered to infants with clinical suspicion of invasive infection. (DOCX)

S6 Table Mortality rate in relation with pathogens identified by hemoculture or quantitative polymerase chain reaction in blood. (DOCX)
