# Supplementary material for: Etiology of severe invasive infections in young infants in rural settings in sub-Saharan Africa
Source: PLoS One. 2022 Feb 25;17(2):e0264322. doi: 10.1371/journal.pone.0264322 (PMC8880396; doi:10.1371/journal.pone.0264322)
Supplement: S1 Appendix — (PDF) [file pone.0264322.s001.pdf]

S1 Appendix: Pathogens included in the Taqman Array Card panel

Port L	Port R
Acinetobacter baumanii	Acinetobacter baumanii
Cytomegalovirus	Cytomegalovirus
Enterococcus faecalis	Enterococcus faecalis
Enterovirus	Enterovirus
Escherichia coli	Escherichia coli
Haemophilius influenzae	Haemophilius influenzae
HSV 1	HSV 1
HSV 2	HSV 2
Klebsiella oxytoca	Klebsiella oxytoca
Klebsiella pneumoniae	Klebsiella pneumoniae
Listeria monocytogenes	Listeria monocytogenes
Mycobacterium tuberculosis	Mycobacterium tuberculosis
Neisseria meningitidis	Neisseria meningitidis
18S	Bacterial 16S
MS2	MS2
PhHV	PhHV
Plasmodium spp.	Ctx-M
Pseudomonas aeruginosa	Pseudomonas aeruginosa
Salmonella spp.	Salmonella spp.
Staphylococcus aureus	Staphylococcus aureus
Streptococcus agalactiae	Streptococcus agalactiae
Streptococcus pneumoniae	Streptococcus pneumoniae
Streptococcus pyogenes	Streptococcus pyogenes
Ureaplasma	Ureaplasma

Note: The target for detection *of E. coli* also reacts with the *Shigella*
