# Supplementary figures and images for: Etiology of severe invasive infections in young infants in rural settings in sub-Saharan Africa
Source: PLoS One. 2022 Feb 25;17(2):e0264322. doi: 10.1371/journal.pone.0264322 (PMC8880396; doi:10.1371/journal.pone.0264322)

**S1 Fig: Flow chart of surveillance of pregnancies and young infants in community**

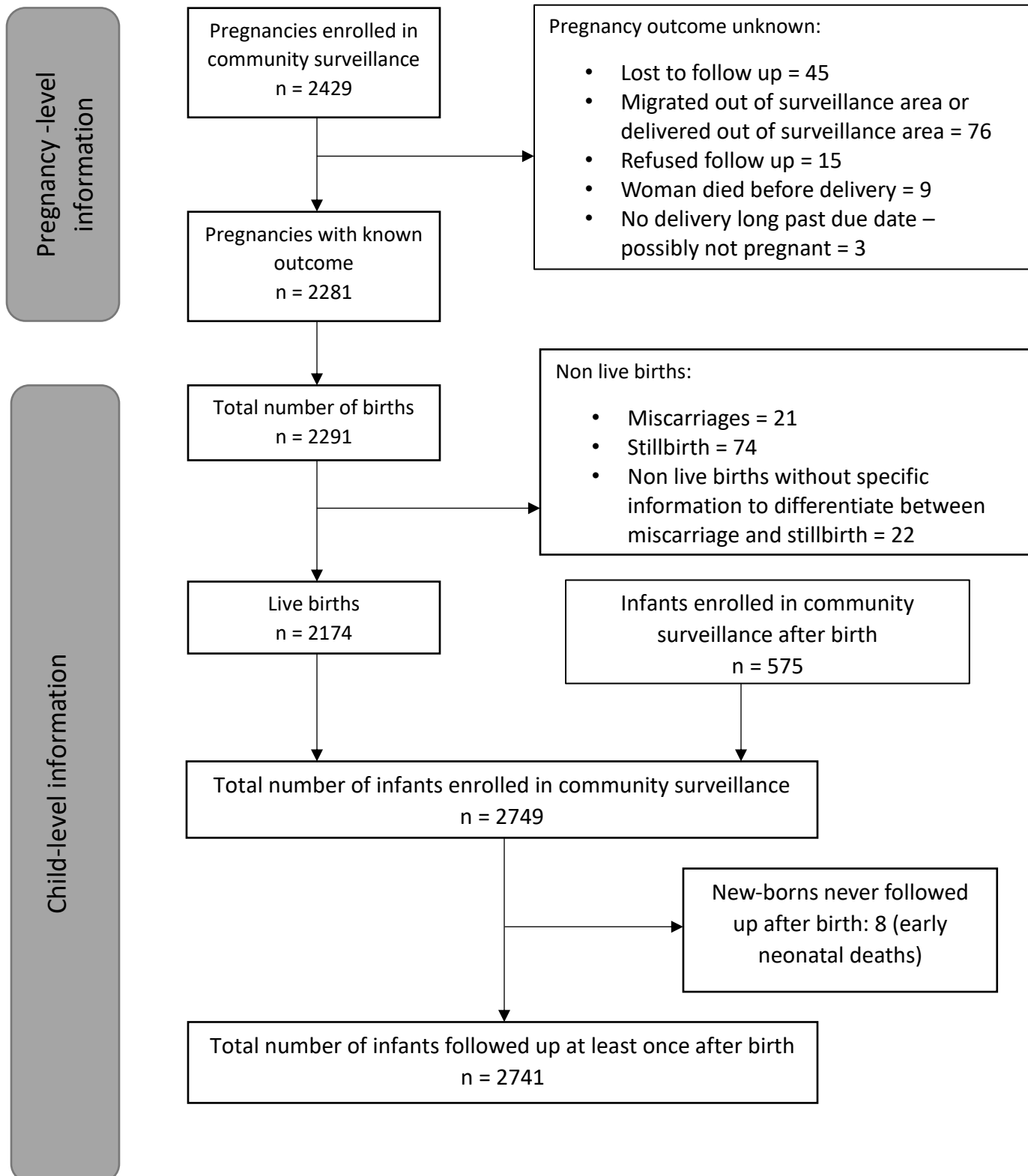

Supplement: S1 Fig — (PDF) [file pone.0264322.s002.pdf]
