# Supplementary material for: Etiology of severe invasive infections in young infants in rural settings in sub-Saharan Africa
Source: PLoS One. 2022 Feb 25;17(2):e0264322. doi: 10.1371/journal.pone.0264322 (PMC8880396; doi:10.1371/journal.pone.0264322)
Supplement: S1 Table — (DOCX) [file pone.0264322.s004.docx]

**S1 Table: List of blood culture isolates determined as clinically non-significant**
