# Supplementary material for: Etiology of severe invasive infections in young infants in rural settings in sub-Saharan Africa
Source: PLoS One. 2022 Feb 25;17(2):e0264322. doi: 10.1371/journal.pone.0264322 (PMC8880396; doi:10.1371/journal.pone.0264322)
Supplement: S4 Table — (DOCX) [file pone.0264322.s007.docx]

**S4 Table: Antibiotic susceptibility and resistance profile of bacteria isolated in hemoculture**

| **Antibiotics** |  | ***Escherichia coli*** | ***Group A streptococcus*** | ***Klebsiella pneumoniae*** | ***Pseudomonas aeruginosa*** | ***Serratia marcescens*** | ***Staphylococcus aureus*** | ***Streptococcus pneumoniae*** | ***Streptococcus sp.*** |
| --- | --- | --- | --- | --- | --- | --- | --- | --- | --- |
|  |  | n=4 | n=1 | n=8 | n=1 | n=1 | n=8 | n=1 | n=1 |
|  |  | n (%) | n (%) | n (%) | n (%) | n (%) | n (%) | n (%) | n (%) |
| **Amikacin** | S | 4 (100) | -- | 8 (100) | 1 (100) | 1 (100) | -- | -- | -- |
|  | I | 0 | -- | 0 | 0 | 0 | -- | -- | -- |
|  | R | 0 | -- | 0 | 0 | 0 | -- | -- | -- |
|  | **Tot.** | **4** | -- | **8** | **1** | 1 | -- | -- | -- |
| **Ampicillin** | S | 1 (25) | 1 (100) | 0 | -- | -- | -- | -- | -- |
|  | I | 0 | 0 | 0 | -- | -- | -- | -- | -- |
|  | R | 3 (75) | 0 | 8 (100) | -- | -- | -- | -- | -- |
|  | **Tot.** | **4** | **1** | **8** | -- | -- | -- | -- | -- |
| **Ciprofloxacin** | S | 3 (75) | -- | 7 (87.5) | 1 (100) | 1 (100) | 2 (100) | -- | -- |
|  | I | 1 (25) | -- | 0 | 0 | 0 | 0 | -- | -- |
|  | R | 0 | -- | 1 (12.5) | 0 | 0 | 0 | -- | -- |
|  | **Tot.** | **4** | -- | **8** | **1** | **1** | **2** | -- | -- |
| **Penicillin** | S | -- | 1 (100) | -- | -- | -- | 0 | 1 (100) | 1 (100) |
|  | I | -- | 0 | -- | -- | -- | 0 | 0 | 0 |
|  | R | -- | 0 | -- | -- | -- | 8 (100) | 0 | 0 |
|  | **Tot.** | -- | **1** | -- | -- | -- | **8** | **1** | **1** |
| **Ceftazidime** | S | 4 (100) | -- | 3 (37.5) | 1 (100) | 1 (100) | -- | -- | -- |
|  | I | 0 | -- | 5 (62.5) | 0 | 0 | -- | -- | -- |
|  | R | 0 | -- | 0 | 0 | 0 | -- | -- | -- |
|  | **Tot.** | **4** | -- | **8** | **1** | **1** | -- | -- | -- |
| **Ceftriaxone** | S | 4 (100) | 1 (100) | 0 | -- | -- | -- | 1 (100) | -- |
|  | I | 0 | 0 | 0 | -- | -- | -- | 0 | -- |
|  | R | 0 | 0 | 8 (100) | -- | -- | -- | 0 | -- |
|  | **Tot.** | **4** | **1** | **8** | -- | -- | -- | **1** | -- |
| **Amoxicillin + Acid clavulanic** | S | 2 (66.7) | -- | -- | -- | -- | -- | -- | -- |
|  | I | 1 (33.3) | -- | -- | -- | -- | -- | -- | -- |
|  | R | 0 | -- | -- | -- | -- | -- | -- | -- |
|  | **Tot.** | **3** | -- | -- | -- | -- | -- | -- | -- |
| **Erythromycin** | S | -- | 1 (100) | -- | -- | -- | 3 (37.5) | 1 (100) | 1 (100) |
|  | I | -- | 0 | -- | -- | -- | 2 (25) | 0 | 0 |
|  | R | -- | 0 | -- | -- | -- | 3 (37.5) | 0 | 0 |
|  | **Tot.** | -- | **1** | -- | -- | -- | **8** | **1** | **1** |
| **Cefoxitin** | S | 4 (100) | -- | 8 (100) | -- | 1 (100) | 8 (100) | -- | -- |
|  | I | 0 | -- | 0 | -- | 0 | 0 | -- | -- |
|  | R | 0 | -- | 0 | -- | 0 | 0 | -- | -- |
|  | **Tot.** | **4** | -- | **8** | -- | **1** | **8** | -- | -- |
| **Norfloxacin** | S | -- | -- | -- | 1 (100) | -- | 2 (100) | -- | -- |
|  | I | -- | -- | -- | 0 | -- | 0 | -- | -- |
|  | R | -- | -- | -- | 0 | -- | 0 | -- | -- |
|  | **Tot.** | -- | -- | -- | **1** | -- | **2** | -- | -- |
| **Nitrofurantoin** | S | -- | -- | -- | -- | -- | 2 (100) | -- | -- |
|  | I | -- | -- | -- | -- | -- | 0 | -- | -- |
|  | R | -- | -- | -- | -- | -- | 0 | -- | -- |
|  | **Tot.** | -- | -- | -- | -- | -- | **2** | -- | -- |
| **Gentamicin** | S | 4 (100) | -- | 0 | 1 (100) | 0 | 8 (100) | -- | -- |
|  | I | 0 | -- | 0 | 0 | 0 | 0 | -- | -- |
|  | R | 0 | -- | 8 (100) | 0 | 1 (100) | 0 | -- | -- |
|  | **Tot.** | **4** | -- | **8** | **1** | **1** | **8** | -- | -- |
| **Nalidixic** **Acid** | S | 1 (100) | -- | 8 (100) | -- | 1 (100) | -- | -- | -- |
|  | I | 0 | -- | 0 | -- | 0 | -- | -- | -- |
|  | R | 0 | -- | 0 | -- | 0 | -- | -- | -- |
|  | **Tot.** | **1** | -- | **8** | -- | **1** | -- | -- | -- |
| **Chloramphenicol** | S | 4 (100) | 1 (100) | 0 | -- | 1 (100) | 8 (100) | 1 (100) | -- |
|  | I | 0 | 0 | 0 | -- | 0 | 0 | 0 | -- |
|  | R | 0 | 0 | 8 (100) | -- | 0 | 0 | 0 | -- |
|  | **Tot.** | **4** | **1** | **8** | -- | **1** | **8** | **1** | -- |
| **Clindamycin** | S | -- | 1 (100) | -- | -- | -- | 2 (100) | 1 (100) | -- |
|  | I | -- | 0 | -- | -- | -- | 0 | 0 | -- |
|  | R | -- | 0 | -- | -- | -- | 0 | 0 | -- |
|  | **Tot.** | -- | **1** | -- | -- | -- | **2** | **1** | -- |
| **Sulfotrim** | S | 1 (25) | -- | 0 | -- | 0 | 2 (100) | 0 | -- |
|  | I | 0 | -- | 0 | -- | 0 | 0 | 1 (100) | -- |
|  | R | 3 (75) | -- | 8 (100) | -- | 1 (100) | 0 | 0 | -- |
|  | **Tot.** | **4** | -- | **8** | -- | **1** | **2** | **1** | -- |
| **Azithromycin** | S | -- | 1 (100) | -- | -- | -- | -- | 1 (100) | -- |
|  | I | -- | 0 | -- | -- | -- | -- | 0 | -- |
|  | R | -- | 0 | -- | -- | -- | -- | 0 | -- |
|  | **Tot.** | -- | **1** | -- | -- | -- | -- | 1 | -- |
| **Cefepime** | S | -- | -- | -- | 1 (100) | -- | -- | -- | -- |
|  | I | -- | -- | -- | 0 | -- | -- | -- | -- |
|  | R | -- | -- | -- | 0 | -- | -- | -- | -- |
|  | **Tot.** | -- | -- | -- | **1** | -- | -- | -- | -- |
| **Tobramycin** | S | -- | -- | -- | 1 (100) | -- | -- | -- | -- |
|  | I | -- | -- | -- | 0 | -- | -- | -- | -- |
|  | R | -- | -- | -- | 0 | -- | -- | -- | -- |
|  | **Tot.** | -- | -- | -- | **1** | -- | -- | -- | -- |
| **Carbapenem†** | S | 4 (100) | -- | 8 (100) | 1 (100) | 1 (100) | -- | 1 (100) | -- |
|  | I | 0 | -- | 0 | 0 | 0 | -- | 0 | -- |
|  | R | 0 | -- | 0 | 0 | 0 | -- | 0 | -- |
|  | **Tot.** | **4** | -- | **8** | **1** | **1** | -- | **1** | -- |
| **Aztreonam** | S | 3 (100) | -- | -- | 1 (100) | -- | -- | -- | -- |
|  | I | 0 | -- | -- | 0 | -- | -- | -- | -- |
|  | R | 0 | -- | -- | 0 | -- | -- | -- | -- |
|  | **Tot.** | **3** | -- | -- | **1** | -- | -- | -- | -- |
| **Cefazolin** | S | 2 (66.7) | -- | -- | -- | -- | -- | -- | -- |
|  | I | 0 | -- | -- | -- | -- | -- | -- | -- |
|  | R | 1 (33.3) | -- | -- | -- | -- | -- | -- | -- |
|  | **Tot.** | **3** | -- | -- | -- | -- | -- | -- | -- |
| **Piperacillin** | S | -- | -- | -- | 1 (100) | -- | -- | -- | -- |
|  | I | -- | -- | -- | 0 | -- | -- | -- | -- |
|  | R | -- | -- | -- | 0 | -- | -- | -- | -- |
|  | **Tot.** | -- | -- | -- | **1** | -- | -- | -- | -- |
| **Piperacillin/Tazobactam** | S | -- | -- | -- | 1 (100) | -- | -- | -- | -- |
|  | I | -- | -- | -- | 0 | -- | -- | -- | -- |
|  | R | -- | -- | -- | 0 | -- | -- | -- | -- |
|  | **Tot.** | -- | -- | -- | **1** | -- | -- | -- | -- |
| **Tetracycline** | S | -- | 0 | -- | -- | -- | 0 | 0 | 0 |
|  | I | -- | 0 | -- | -- | -- | 0 | 0 | 0 |
|  | R | -- | 1 (100) | -- | -- | -- | 1 (100) | 1 (100) | 1 (100) |
|  | **Tot.** | -- | **1** | -- | -- | -- | **1** | **1** | **1** |

*S: susceptible, R: resistant, I: intermediate, Tot.: total number of isolated tested for this antibiotic.*

*% are expressed as % of total of total number of isolated tested for the antibiotics*

*†: Results available for imipenem, meropenem, and ertapenem have been grouped together under Carbapenem*
