# Supplementary material for: Etiology of severe invasive infections in young infants in rural settings in sub-Saharan Africa
Source: PLoS One. 2022 Feb 25;17(2):e0264322. doi: 10.1371/journal.pone.0264322 (PMC8880396; doi:10.1371/journal.pone.0264322)
Supplement: S5 Table — (DOCX) [file pone.0264322.s008.docx]

**S5 Table: Frequency of different systemic antibiotics administered to infants with clinical suspicion of invasive infection**

* Out of the 634 infants enrolled, a total of 44 infants didn’t received systemic antibiotics.
