# Supplementary material for: Etiology of severe invasive infections in young infants in rural settings in sub-Saharan Africa
Source: PLoS One. 2022 Feb 25;17(2):e0264322. doi: 10.1371/journal.pone.0264322 (PMC8880396; doi:10.1371/journal.pone.0264322)
Supplement: S6 Table — (DOCX) [file pone.0264322.s009.docx]

**S6 Table: Mortality rate in relation with pathogens identified by hemoculture or quantitative polymerase chain reaction in blood**
